# Fatal Babesiosis in Man, Finland, 2004

**DOI:** 10.3201/eid1607.091905

**Published:** 2010-07

**Authors:** Karita Haapasalo, Pekka Suomalainen, Antti Sukura, Heli Siikamäki, T. Sakari Jokiranta

**Affiliations:** Author affiliations: University of Helsinki, Helsinki, Finland (K. Haapasalo, A. Sukura, T.S. Jokiranta);; Hospital District of Helsinki and Uusimaa, Helsinki (K. Haapasalo, T.S. Jokiranta);; South Karelia Central Hospital, Lappeenranta, Finland (P. Suomalainen);; Helsinki University Central Hospital, Helsinki (H. Siikamäki)

**Keywords:** Babesiosis, Babesia divergens, Ixodes, tick-borne diseases, vector-borne infections, parasites, Finland, dispatch

## Abstract

We report an unusual case of human babesiosis in Finland in a 53-year-old man with no history of splenectomy. He had a rudimentary spleen, coexisting Lyme borreliosis, exceptional dark streaks on his extremities, and subsequent disseminated aspergillosis. He was infected with *Babesia divergens*, which usually causes bovine babesiosis in Finland.

Babesiosis is an arthropod-transmitted infection caused by an apicomplexan parasite. Most zoonotic cases in humans have been reported from the eastern coast of the United States, where the causative agent is *Babesia microti*, which is transmitted from white-footed mice to humans by *Ixodes scapularis* ticks (*1**,**2*). Rare cases of human babesiosis caused by *B*. *divergens* have been reported in Europe. *B*. *divergens* is a bovine parasite transmitted mainly by *I. ricinus* ticks. Both *Ixodes* spp. ticks also transmit *Borrelia* *burgdorferi*, the etiologic agent of Lyme borreliosis. In certain areas of the United States, >10% of patients with a diagnosis of Lyme disease are co-infected with *B. microti* (*3*). Co-infections with *Borrelia* spp. and *B*. *divergens* infections have been documented only serologically (*4*).

*Babesia* sporozoites are transmitted to the vertebrate host by a tick bite (*5*). The sporozoites invade erythrocytes and transform into ring-form trophozoites and typical Maltese cross assemblies of merozoites. Parasites lyse infected erythrocytes, which release merozoites that can invade new erythrocytes (*6**,**7*). Trophozoites can alternatively develop into gametocytes, enabling continuation of the life cycle in the tick after it has had a blood meal.

Most disease manifestations of human babesiosis are related to hemolysis (*1*). Symptoms are anemia, malaise, fever, chills, myalgia, and fatigue. High parasitemia levels can cause massive hemoglobinuria, acute renal tubular necrosis, and renal failure. In addition to hemolysis-associated manifestations, acute respiratory distress syndrome may occur as a complication and lead to death (*8*). *B*. *divergens* infections in Europe have been severe, and the mortality rate is high (42%) compared with that of *B. microti* infections in the United States (5%–20%) (*1**,**2**,**9*). All 22 published cases of *B. divergens* infection in Europe have occurred in patients who have undergone splenectomy (*2*). We report an unusual case of human babesiosis in Finland.

## The Patient

A 53-year-old man was admitted to South Karelia Central Hospital in Lappeenranta, Finland, in September 2004 with septic shock. The patient had a history of severe alcohol-induced pancreatitis (in 1993), subsequent type 1 diabetes mellitus, *Staphylococcus aureus* septicemia (in 2000), and atherosclerosis. He had had fever, back pain, and fatigue for a week before admission and reported that his urine was darker and lower in volume than usual for several days. The patient had no history of travel abroad in the past 5 years.

At admission, the patient was conscious. Physical examination and intensive care unit monitoring indicated septic shock and lactate acidosis. The Physiology and Chronic Health Evaluation score of the patient was 60%, and the Simplified Acute Physiology score was 50%. The patient was treated with routine intensive care measures, which were started at admission.

**Figure 1:**
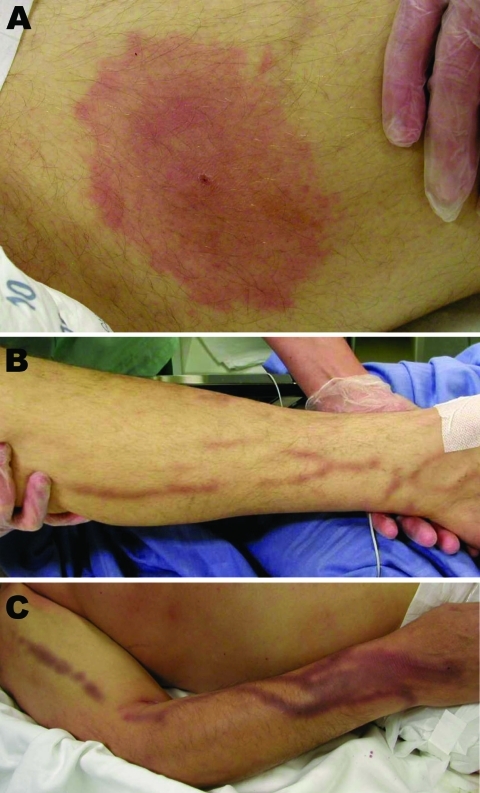
Lesions of the patient infected with *Babesia divergens* 1 day after hospitalization, Finland, 2004. A) Left thigh showing a classical erythema chronicum migrans lesion; B) left leg and C) right arm showing dark purple streaks.

**Figure 2:**
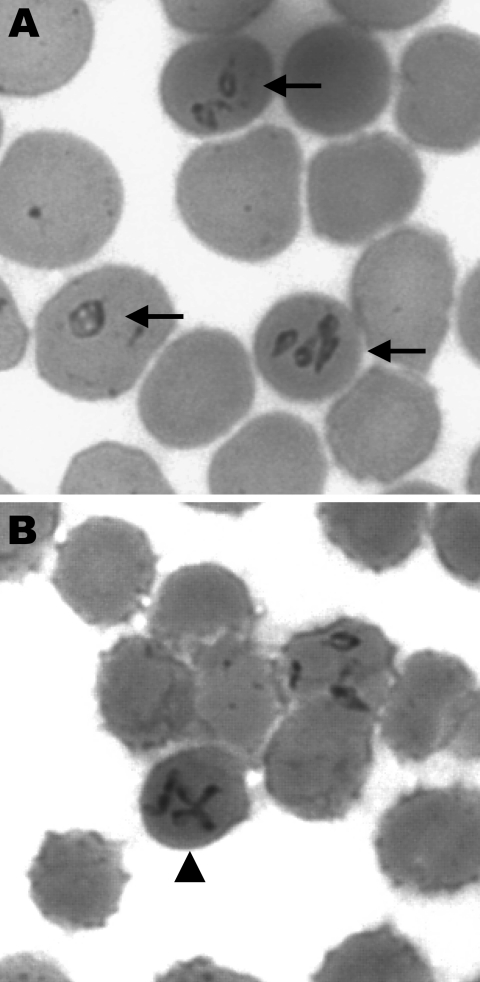
Giemsa-stained smears of peripheral blood of the patient infected with *Babesia divergens*, Finland, 2004. Intraerythrocytic parasites are indicated by arrows (A), and a representative Maltese cross form of the parasite is indicated by an arrowhead (B). (Original magnification ×750.)

Physical examination showed a wide (maximum diameter 10 cm) erythema chronicum migrans (ECM) lesion on the left thigh (Figure 1, panel A) and dark streaks on both legs and the right arm (Figure 1, panels B, C). During the first 24 hours, laboratory analyses showed massive hemolysis (plasma hemoglobin 2,650 mg/dL, blood hemoglobin 5.7 g/dL, haptoglobin 30 mg/dL, lactic dehydrogenase 11,240 U/L, bilirubin 213 µmol/L), thrombocytopenia (thrombocyte count 56,000/µL), an elevated C-reactive protein level (13.9 mg/dL at admission), and an elevated aspartate aminotransferase level (9,559 U/L). Ultrasound showed a low signal from the spleen. Microscopic examination of a blood smear was conducted 25 hours after admission because of suspicion of hemolytic uremic syndrome and showed intraerythrocytic structures typical of babesiosis with a parasitemia level of ≈10% (Figure 2).

The patient was treated with intravenous quinine (1,000 mg 3×/d for 12 days), clindamycin (600 mg 4×/d for 12 days), and cefotaxime (2 g/d for 12 days). At the end of day 2, a 9-liter blood exchange was performed. Despite these procedures and reduction of the high level of parasitemia (to 1% within 2 days), the patient had hypotension and was treated in the intensive care unit in a respirator under propofol-based sedation. At day 9, he had pulmonary aspergillosis (infection with *Aspergillus fumigatus*), *S*. *epidermidis* in blood cultures, and *Candida albicans* cultured from the tip of his dialysis catheter. After the patient received a diagnosis of multiple organ failure, treatment was terminated on day 12; he died the same day. Histologic analysis of autopsy specimens showed aspergillosis in the lungs, heart, and kidneys. The spleen was rudimentary, and no malignancy was found. The patient was HIV negative.

To identify *Babesia* spp., DNA was extracted from anticoagulated venous blood samples obtained on day 2. DNA was amplified and the PCR product was sequenced as described (*10*). The sequence obtained (GenBank accession no. GU945501) was 100% identical to previously a reported *B*. *divergens* 18S rDNA sequence (GenBank accession no. AY789076.1).

## Conclusions

We report an unusual human case of babesiosis and provide molecular evidence that the causative organism was *B*. *divergens*. Human babesiosis has not been previously reported in Finland. Eight fatal cases of babesiosis have been described in Europe (1 each in the former Soviet Union, France, Spain, and the former Yugoslavia, and 4 in the British Isles) (*2**,**11*). All cases were *B*. *divergens* infections in patients who had undergone splenectomy. This species has been identified by inoculation into animals or by serologic analysis.

**Figure A1:**
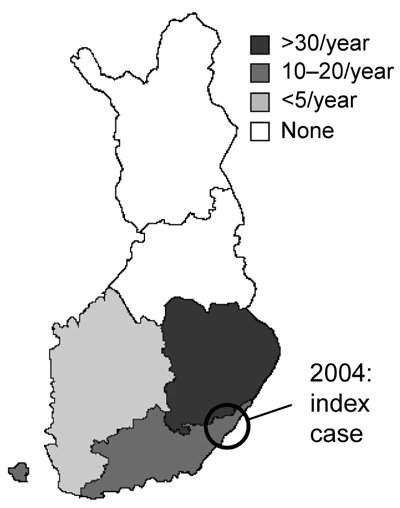
Municipality in Finland where human babesiosis infection (index case) was acquired and 3 bovine babesiosis cases were found in 2004. Average annual number of cases of babesiosis in cattle during 1997–2003 is indicated.

Bovine babesiosis is endemic to Finland, although its incidence has dramatically decreased over the past 2 decades. Monthly data for bovine babesiosis in Finland are obtained by municipal veterinarians who send reports to the Ministry of Agriculture and Forestry (T. Aaltonen, unpub. data). Three cases of bovine babesiosis were reported to health authorities in the municipality where the patient lived in the same year he was infected and became ill (Figure A1). The patient lived in the country next to a cattle farm. He took walks and worked in the forest nearby. Because the patient had no travel history, we conclude that babesiosis was acquired in Finland, and, most likely, locally from the zoonotic reservoir of *B*. *divergens* in cattle through a tick bite (Table). This conclusion is supported by the presence of ECM and a tick bite on the thigh of the patient. Infection with *Babesia* sp. and *Borrelia* sp. from the same tick bite is likely because ECM and babesiosis caused by *B*. *divergens* are manifested 3–30 days and 7–21 days, respectively, after a tick bite.

**Table:** Incidence of cattle babesiosis, Finland*

District	Year						
	1965	1975	2000	2002	2002	2003	2004
Southern	–	–	21	7	23	6	11
Eastern†	–	–	39	31	27	28	18
Oulu	–	–	0	0	0	0	0
Lapland	–	–	0	0	0	0	0
Ahvenanmaa	–	–	18	12	18	2	0
Western	–	–	1	1	6	6	2
Total	4,796	2,524	79	51	74	42	312

*–, no district data were available. †Region where the index case-patient lived.

The poor outcome of the patient can be explained by several factors. His immune system was not intact. He also had diabetes, which impaired his general immunity. Although he had not undergone splenectomy, he was also considered asplenic because only a rudimentary spleen was found by autopsy. The rudimentary spleen may have been caused by severe pancreatitis or by high consumption of alcohol (*12*). Invasive aspergillosis, which is usually seen only in severely immunocompromised patients, also developed in the patient. The origin of dark streaks on his arms and legs is not clear but was probably caused by massive intravascular hemolysis, which was also suggested by dark urine.

Co-infection with *Babesia* sp. and *Borrelia* sp. has been shown to cause disease with additional symptoms and a more persistent illness than infection with *B*. *microti* (*3*). This co-infection could have caused the severe and fatal disease in our patient. Thus, clinicians should be aware of co-infection with *B*. *divergens* and *Borrelia* sp.
